# 5′-tiRNA-Lys maintains intestinal epithelial homeostasis by EWSR1-dependent suppression of miR-125a and autophagy activation: 5′-tiRNA-Lys modulates autophagy in IECs

**DOI:** 10.3724/abbs.2025074

**Published:** 2025-06-23

**Authors:** Ningqin Xu, Ju Huang, Yiming Wang, Yaxin Liu, Yangwei Jiang, Wei Zhou, Jinghao Sheng, Lahong Zhang

**Affiliations:** 1 School of Clinical Medicine Hangzhou Normal University Hangzhou 310015 China; 2 Liangzhu Laboratory Zhejiang University Hangzhou 311121 China; 3 Department of General Surgery Sir Run Run Shaw Hospital Zhejiang University School of Medicine Hangzhou 310058 China; 4College of Life Sciences Zhejiang University Hangzhou 310058 China; 5 Laboratory Department Hangzhou Normal University Affiliated Hospital Hangzhou 310015 China

**Keywords:** inflammatory bowel disease, autophagy, intestinal epithelial cell, tRNA-derived small RNA, 5′-tiRNA-Lys

## Abstract

The intestinal epithelium relies on autophagy to maintain barrier integrity and homeostasis, and dysregulation of this process has been implicated in inflammatory bowel disease (IBD). While tRNA-derived small RNAs (tsRNAs) are emerging as regulators of cellular stress responses, their roles in intestinal autophagy remain poorly understood. Here, we identify that 5′-tiRNA-Lys, a tsRNA derived from mature tRNA-Lys, is significantly upregulated in the inflamed intestinal epithelium of IBD patients. Overexpression of 5′-tiRNA-Lys in mice ameliorates dextran sulfate sodium (DSS)-induced colitis. Mechanistically, 5′-tiRNA-Lys enhances autophagy in intestinal epithelial cells (IECs), as evidenced by elevated LC3-II level and autophagosome formation. RNA pull-down and immunoprecipitation assays reveal that 5′-tiRNA-Lys directly binds to the RNA-binding protein EWSR1 via its RNA recognition motif, disrupting EWSR1’s interaction with the Drosha/DGCR8 microprocessor complex. This interference specifically suppresses the maturation of miR-125a, a microRNA that targets the autophagy-promoting gene *UVRAG*. Consequently, 5′-tiRNA-Lys increases UVRAG expression, thereby enhancing autophagic activity. Our findings reveal that 5′-tiRNA-Lys modulates autophagy through EWSR1-mediated miR-125a processing, which in turn affects intestinal inflammation, highlighting the potential of 5′-tiRNA-Lys as a therapeutic target for IBD.

## Introduction

The intestinal epithelium is a critical interface between the host and the external environment, acting as a selective barrier that facilitates nutrient absorption while simultaneously preventing the entry of pathogens [ 1, 2] . The integrity and function of this barrier are essential for maintaining intestinal homeostasis [3]. Autophagy, an evolutionarily conserved intracellular recycling process, plays a pivotal role in intestinal epithelial cell (IEC) biology [ 4, 5] . It is involved in protecting against enteric pathogens [6], maintaining the homeostatic and secretory capacity of specialized IECs, such as Paneth and goblet cells [ 7, 8] , and supporting the survival of intestinal stem cells [9]. Accumulating evidence from genome-wide association studies (GWASs) and animal models shows that disruptions in autophagy are associated with various gastrointestinal disorders, particularly inflammatory bowel disease (IBD) [ 10– 12] . This highlights the crucial role of autophagy in maintaining gut health and underscores the need for a deeper understanding of its mechanisms and implications in the context of intestinal homeostasis and disease.

Transfer RNA (tRNA)-derived small RNAs (tsRNAs) are a class of non-coding RNAs that play critical roles in maintaining tissue homeostasis [ 13, 14] . These tsRNAs can be broadly classified into two main categories: tRNA-derived stress-induced RNAs (tiRNAs) and tRNA-derived fragments (tRFs) [15]. tiRNAs are generated through the cleavage of mature tRNAs at the anticodon loop by angiogenin (ANG) under various cellular stress conditions, such as oxidative stress, heat shock, or nutrient deprivation [ 16– 19] . These tiRNAs, which typically range from 30 to 40 nucleotides in length, have been shown to regulate cell survival and apoptosis by modulating the expressions of stress response genes and interacting with the translation machinery [ 20, 21] . On the other hand, tRFs are produced through the cleavage of mature or precursor tRNAs at various positions, giving rise to distinct subtypes, such as 5′-tRFs, 3′-tRFs, and i-tRFs (internal tRFs). tRFs, which are usually 14 to 30 nucleotides long, have been implicated in a wide range of biological processes, including gene regulation, cell proliferation, and differentiation [ 22, 23] .

The main functional mechanisms of tsRNAs within cells involve their interactions with various cellular components. For example, tsRNAs can bind to messenger RNAs (mRNAs) and regulate their stability or translation efficiency, thereby modulating gene expression [ 24, 25] . Additionally, tsRNAs have been shown to interact with RNA-binding proteins (RBPs) and influence their function, which can impact diverse cellular processes, such as RNA splicing, localization, and degradation [ 26– 28] . Recent studies from our group revealed that tiRNAs generated by ANG-mediated cleavage play a protective role in intestinal epithelial cell survival during intestinal inflammation [ 29, 30] . However, the precise regulatory mechanisms by which tiRNAs exert their effects on intestinal epithelial cells remain unclear and warrant further investigation.

Given the emerging evidence of the involvement of tsRNAs in various cellular processes and their potential roles in maintaining tissue homeostasis, particularly in the context of intestinal inflammation, understanding their functional mechanisms is crucial. Thus, we first analyzed the expression profile of tsRNAs in the intestinal epithelial tissue of patients with IBD and identified 5′-tiRNA-Lys as a significantly upregulated tsRNA. We further explored its functional role in a colitis mouse model and investigated the underlying molecular mechanism, particularly how 5′-tiRNA-Lys affects autophagy and miRNA processing. Our results provide new insights into the pathogenesis of IBD and reveal the value of 5′-tiRNA-Lys as a potential IBD therapeutic target.

## Materials and Methods

### Human samples

Intestinal tissue samples were collected from patients diagnosed with inflammatory bowel disease (IBD) at the IBD Center of Sir Run Run Shaw Hospital, affiliated with Zhejiang University School of Medicine, as previously described [ 30, 31] . All sample collection procedures were conducted in accordance with ethical standards. Total RNA was extracted from the frozen tissue samples via TRIzol Reagent (#15596018; Thermo Fisher Scientific, Waltham, USA) according to the manufacturer’s protocol. The quality and concentration of the extracted RNA were assessed using a NanoDrop 2000 spectrophotometer (Thermo Fisher Scientific). RNA was then used for various downstream analyses, including polyacrylamide gel electrophoresis (PAGE), Northern blot analysis, quantitative real-time PCR (qPCR), and high-throughput RNA sequencing, to profile tRNA-derived small RNAs (tsRNAs).

### RNA PAGE and Northern blot analysis

For PAGE and Northern blot analysis, 3 μg of total RNA was denatured in 1× denaturing buffer at 70°C for 5 min and then resolved on 15% urea-PAGE gels using standard electrophoresis conditions. Following electrophoresis, RNA was transferred to a positively charged nylon membrane (#RPN303B; GE Healthcare, Chicago, USA) by electroblotting via a semidry transfer system. The membranes were crosslinked by UV irradiation. The membranes were then pre-hybridized in DIG Easy Hyb buffer (#11796895001; Roche, Basel, Switzerland) for 1 h at 42°C to block non-specific binding sites. Probes complementary to the 5′-tiRNA-Lys and 5′-tiRNA-Ctrl sequences were synthesized and labelled with DIG ( Supplementary Table S1). Hybridization was carried out overnight at 42°C with gentle agitation to ensure optimal probe binding. Following hybridization, the membranes were washed three times with 2× SSC containing 0.1% SDS for 15 min at 42°C, followed by two washes with 0.1× SSC containing 0.1% SDS for 15 min at room temperature to remove non-specifically bound probes. Detection was performed via a DIG Luminescent Detection kit (#11363514910; Roche) according to the manufacturer’s instructions. Briefly, the membranes were incubated with an anti-DIG-AP antibody (#11093274910; Roche) diluted 1:10,000 in DIG Wash and Block Buffer for 1 h at room temperature. After washing, the membranes were incubated with CSPD substrate (#11755633001; Roche) for 5 min to develop the chemiluminescent signal. The signals were detected and imaged using an Imager 680 System (Amersham, Buckinghamshire, UK).

### Quantitative real-time PCR (qPCR)

qPCR was conducted using the TaqMan Assay kit (GenePharma, Shanghai, China) with custom-designed stem-loop primers for 5′-tiRNA-Lys, 5′-tiRNA-Val, miRNA-125a, miRNA-152, miRNA-20a, miRNA-181d, and *U6* small nuclear RNA (as an internal control). For each target RNA, specific stem-loop primers were custom designed and validated to ensure optimal amplification efficiency. The expression levels of pri-miRNA-125a and pre-miRNA-125a were quantified using the TaqMan pri-miRNA and pre-miRNA assay kits (#4427012 and #4426961, respectively; Thermo Fisher Scientific). These assays utilize specific primers and probes designed to target the primary and precursor forms of miRNA-125a, enabling accurate differentiation and quantification of each transcript. All the assays were performed on a Roche LightCycler 480 Real-Time PCR Detection System (Roche). Relative expression levels of the target RNAs were calculated using the 2 ^−ΔΔCt^ method, with *U6* small nuclear RNA serving as the endogenous control for normalization. The sequences of the primers and probes are listed in Supplementary Table S2.

### Small RNA sequencing and analysis

Small RNA sequencing was carried out by Kangchen Biotech (Shanghai, China). Briefly, total RNA was separated on a 15% PAGE gel, and the 10–50 nt fragments were excised and recovered. Prior to library construction, several pretreatments were conducted to facilitate adapter ligation and reverse transcription by rtStar™ tRF&tiRNA Pretreatment kit (#AS-FS-005; Arraystar, Rockville, USA). Library preparation was performed using the NEBNext Small RNA Library Prep Set for Illumina (#E7330S; NEB, Beverly, USA), and the resulting libraries were validated on an Agilent BioAnalyzer 2100 (Agilent, Santa Clara, USA). Sequencing on an Illumina HiSeq 2500 platform (Illumina, San Diego, USA) generated 50-bp single-end reads. Adapter trimming and quality control were performed using standard tools such as Cutadapt, followed by FastQC. For the tsRNAs, the reads were identified and annotated using MINTmap. MicroRNA (miRNA) analysis involves mapping to the human reference genome, annotation with miRBase, and differential expression analysis using DESeq2. Expression profiles were compared across samples, and significantly altered small RNAs were subjected to further investigations.

### Animal studies and DSS-induced colitis model

Male C57BL/6 mice (6–8 weeks old) were purchased from GemPharmatech (Nanjing, China), housed under specific pathogen-free (SPF) conditions with a 12/12-h light/dark cycle at the Laboratory Animal Centre of Zhejiang University, and provided with water and a standard laboratory diet ad libitum. All animal experiments were approved by the Medical Experimental Animal Care Commission of Zhejiang University (#ZJU20220219).

To overexpress 5′-tiRNA-Lys (or a control fragment, 5′-tiRNA-Ctrl), a custom AAV9 vector was generated by GenePharmatech. The mice were fasted overnight and pre-treated with 20 mM N-acetyl-L-cysteine (NAC; a mucolytic agent for the intestinal mucosal surface; Sigma-Aldrich, St Louis, USA). Briefly, the mouse colon was washed with an intrarectal injection of 300 μL of 20 mM NAC using a stainless steel straight round-tip microsyringe and allowed to drain without sedation for 30 min. Then, the mice were anaesthetized with inhaled isoflurane, and 1 × 10 ^11^ vector genomes (vg) in 100 μL of PBS were given through an enema. After 21 days of AAV infection, these mice were subjected to DSS administration. To establish a DSS-induced colitis model, the mice were given 2.5% (w/v) DSS (molecular weight: 36–50 kDa; #160110; MP Biomedicals, Santa Ana, USA) in their drinking water for 6 days, followed by 3 days of regular water. Clinical parameters—including weight loss, stool consistency, and rectal bleeding—were recorded daily to calculate the disease activity index (DAI) [32]. At the endpoint, the mice were euthanized, and colonic tissues were collected for histological and molecular analyses.

### Hematoxylin and eosin (H&E) staining

Colonic tissues were fixed in 4% paraformaldehyde overnight at 4°C, dehydrated and embedded in paraffin. The sections were deparaffinized in xylene for 10 min and rehydrated through a graded ethanol series (95%, 90%, 80%, and 70%) for 5 min each. They were stained with hematoxylin (#C0107; Beyotime, Shanghai, China) for 6 min, differentiated in 5% acetic acid for 30 s, and treated with a bluing solution to restore the blue color of the nuclei. Eosin (#C0109; Beyotime) was then applied for 2 min. After staining, the sections were dehydrated through increasing concentrations of ethanol (70%, 80%, 90%, and 95%) for 5 min each and cleared in xylene for 5 min. Finally, they were air-dried, mounted, and observed using a Leica DM2000 LED microscope (Leica, Wetzlar, Germany). Histopathological scoring was performed by two blinded investigators using a standardized scoring system.

### Inflammatory cytokine detection

To quantify the levels of CCL3, CXCL1, IL-6, IL-1β, and TNFα in colonic tissue samples, the corresponding enzyme-linked immunosorbent assay (ELISA) kits were used following the manufacturers’ protocols. Colonic tissues were homogenized in lysis buffer supplemented with protease and phosphatase inhibitors (#P8340; Sigma-Aldrich) to prevent protein degradation. The homogenates were centrifuged at 12,000 *g* for 10 min at 4°C, and the supernatants were collected for subsequent analyses. Cytokine levels were determined using the following commercially available ELISA kits: CCL3 (#KE10023; Proteintech, Wuhan, China), CXCL1 (#KE10104; Proteintech), IL-6 (#KE10007; Proteintech), IL-1β (#MLB00C; R&D Systems, Minneapolis, USA), and TNFα (#KE10002; Proteintech). For each cytokine, a standard curve was constructed with the provided standards, and sample dilutions were optimized to ensure measurements within the linear range. The assays were performed in 96-well plates, with all samples run in triplicate. Finally, the measured cytokine concentrations were normalized to the total protein content of the tissue samples, which was determined using a BCA protein assay kit (#P0012; Beyotime). All procedures were executed under rigorous quality control to ensure the reliability of the data.

### Cell culture and transfection

The human colonic epithelial cell line NCM460 (#CL0393; Hunan Fenghui Biotechnology, Changsha, China) was maintained in RPMI 1640 medium (#11875093; Thermo Fisher Scientific) supplemented with 10% fetal bovine serum (FBS; #26010074; Thermo Fisher Scientific) and 1% (v/v) penicillin-streptomycin (#15140122; Thermo Fisher Scientific). The cells were cultured at 37°C in a humidified incubator with 5% CO _2_. Subculturing was performed every 2–3 days to maintain logarithmic growth.

For transfection, 5′-tiRNA-Lys or 5′-tiRNA-Ctrl (both chemically synthesized by Takara, Dalian, China; Supplementary Table S3) was introduced into NCM460 cells using Lipofectamine RNAiMIX (#13778075; Thermo Fisher Scientific) according to the manufacturer’s instructions. Briefly, cells were seeded in 6-well plates the day before transfection to achieve approximately 70%–80% confluence at the time of transfection. The tiRNAs and Lipofectamine RNAiMIX were each diluted in Opti-MEM (#31985070; Thermo Fisher Scientific), combined, and then added to the cells. The transfection complexes were incubated with the cells for 24–48 h before downstream analyses were performed.

### Immunofluorescence staining

NCM460 cells were seeded on sterile glass coverslips and allowed to adhere until they reached approximately 70%–80% confluence. The cells were then fixed in 4% paraformaldehyde for 15 min, permeabilized with 0.1% Triton X-100, and blocked with 5% bovine serum albumin (BSA) for 1 h. After being blocked, the coverslips were incubated overnight at 4°C with an anti-LC3 primary antibody (#3868; Cell Signaling Technology, Danvers, USA) diluted 1:200 in PBS containing 1% BSA. After three washes in PBS, the cells were incubated for 1 h at room temperature in the dark with an Alexa Fluor 555-conjugated secondary antibody (#A31572; Thermo Fisher Scientific) and then washed again in PBS. The nuclei were counterstained with DAPI for 5 min, and coverslips were mounted on glass slides using antifade mounting medium. Images were acquired via a Nikon A1 confocal microscope (Nikon, Tokyo, Japan) with appropriate excitation and emission settings.

### Western blot analysis

Cells or tissues were lysed in RIPA buffer (50 mM Tris-HCl, pH 7.4; 150 mM NaCl; 1% NP-40; 0.5% sodium deoxycholate; and 0.1% SDS) supplemented with protease inhibitors (#04693159001; Roche). The total protein concentration was determined using a BCA protein assay kit (Beyotime). Equal amounts of protein (approximately 20–40 μg per lane) were separated by SDS-PAGE and transferred onto PVDF membranes (#IPVH00010; Millipore, Billerica, USA). The membranes were blocked in 5% nonfat milk or 5% BSA in TBST (20 mM Tris-HCl, pH 7.5; 150 mM NaCl; and 0.1% Tween-20) for 1 h at room temperature. The samples were then incubated overnight at 4°C with the following primary antibodies: anti-LC3 (#14600-1-AP; Proteintech), anti-GAPDH (#110494-1-AP; Proteintech), anti-EWSR1 (#55191-1-AP; Proteintech), anti-DGCR8 (#60084-1-Ig; Proteintech), anti-Drosha (#55001-1-AP; Proteintech), anti-UVRAG (#29190-1-AP; Proteintech), anti-Flag (#20543-1-AP; Proteintech), and anti-p62/SQSTM1 (#84826-1-RR; Proteintech), each diluted according to the manufacturer’s recommendations. After three washes in TBST, the membranes were incubated with the appropriate horseradish peroxidase (HRP)-conjugated secondary antibodies ( *e*. *g*., #7074 for anti-rabbit or #7076 for anti-mouse; Cell Signaling Technology) at room temperature for 1 h. Finally, the protein bands were visualized using an enhanced chemiluminescence (ECL) detection reagent (#P0018S; Beyotime) and imaged with an Amersham Imager 680 System.

### Biotin-labelled 5′-tiRNA pull-down and mass spectrometry

Biotin-labelled 5′-tiRNA-Lys was synthesized by Takara and transfected into NCM460 cells. After 24 h, the cells were harvested and lysed in mild lysis buffer (20 mM Tris-HCl, pH 7.4; 100 mM KCl; 5 mM MgCl _2_; 0.5% NP-40; and 1 mM DTT). The lysates were clarified by centrifugation, and the supernatant was incubated with streptavidin-coated magnetic beads (#88817; Thermo Fisher Scientific) for 2 h at 4°C. The beads were then washed to remove non-specifically bound proteins. The bound proteins were subsequently eluted. The eluate was submitted to PTM Biolabs for mass spectrometry (LC-MS/MS) analysis. The raw MS data were processed and analyzed using Mascot (Matrix Science, London, UK) for protein identification and quantification.

### RNA immunoprecipitation

NCM460 cells were grown to approximately 70% confluence and transfected with constructs expressing various EWSR1 deletion mutants. After 48 h, the cells were washed with cold PBS and lysed in RIP lysis buffer (25 mM Tris-HCl, pH 7.4; 150 mM KCl; 5 mM EDTA; 0.5% NP-40; and 1 mM DTT) supplemented with protease and RNase inhibitors (#N8080119; Thermo Fisher Scientific). The lysates were clarified by centrifugation at 12,000 *g* for 10 min at 4°C.

For immunoprecipitation, the cleared lysates were incubated overnight at 4°C with either an anti-EWSR1 antibody (Proteintech) or an anti-Flag antibody (Proteintech), both of which were pre-bound to protein A/G magnetic beads (#88802; Thermo Fisher Scientific). Normal IgG served as a negative control. After incubation, the beads were collected and washed several times with RIP wash buffer (the same as lysis buffer but without DTT) to remove non-specifically bound components. Total RNA was then extracted from the immunoprecipitated complexes using TRIzol reagent (Thermo Fisher Scientific). qPCR was subsequently performed to assess the enrichment of 5′-tiRNA-Lys and 5′-tiRNA-Val.

### Co-immunoprecipitation

The cells were harvested and lysed in IP buffer (50 mM Tris-HCl, pH 7.4; 150 mM NaCl; and 1% NP-40). The lysates were incubated overnight at 4°C with the anti-EWSR1 antibody (Proteintech) or normal IgG as a negative control. Protein A/G magnetic beads were then added and incubated for an additional 2 h at 4°C. After extensive washing with IP buffer, the immunoprecipitated complexes were eluted by boiling in SDS sample buffer and subjected to western blot analysis.

### Pri-miR-125a stability assay

To assess whether 5′-tiRNA-Lys affects pri-miR-125a stability, we performed RNA stability assays using actinomycin D (#HY-17559; MedChemExpress, Monmouth Junction, USA). NCM460 cells transfected with 5′-tiRNA-Lys or 5′-tiRNA-Ctrl were treated with actinomycin D (5 μg/mL) to inhibit *de novo* RNA synthesis. The cells were harvested at 0, 1, 2, 4, and 6 h post-treatment. Total RNA was extracted using TRIzol reagent, and qPCR was performed to measure pri-miR-125a levels. The decay rate was calculated by plotting the log-transformed relative pri-miR-125a expression against time. Three independent experiments were performed.

### Prediction of 5′-tiRNA-Lys binding to EWSR1

Protein-RNA complex structure prediction was performed with AlphaFold 3 [33] using the sequences of EWSR1 and 5′-tiRNA-Lys-CTT. To account for structural variability, 20 independent predictions with randomized seeds were performed, yielding 100 structural models (5 models per seed). These models were ranked by AlphaFold 3-provided confidence scores, and the top-ranked model was selected as the putative complex structure with residue-wise confidence metrics. All the structures were visualized via UCSF ChimeraX [34].

### Dual-luciferase reporter assays

The 3′UTR of the *UVRAG* gene containing the predicted miR-125a binding site was cloned and inserted into the *Xho*I and *Not*I restriction sites of the psiCHECK-2 dual-luciferase reporter vector (#C8021; Promega, Madison, USA). A mutant construct (mut), in which the miR-125a seed sequence was disrupted, was generated using the Fast Multisite Mutagenesis System (#FM201-01; TransGen Biotech, Beijing, China). NCM460 cells were co-transfected with either the wild-type (wt) or mutant (mut) 3′UTR reporter plasmids, along with miR-125a mimics, inhibitors, negative controls (synthesized by GenePharma; Supplementary Table S3), or 5′-tiRNA-Lys/5’-tiRNA-Ctrl as indicated, using Lipofectamine 3000 (#L3000001; Thermo Fisher Scientific). At 48 h post-transfection, luciferase activity was measured using the Dual-Luciferase Reporter Assay System (E1910; Promega), as described previously [35]. Firefly luciferase activity was normalized to Renilla luciferase activity in the same well.

### Statistical analysis

All data are presented as the mean ± standard error of the mean (SEM). Statistical analyses were conducted using GraphPad Prism 10.4 (GraphPad Software, La Jolla, USA). Differences between two groups were evaluated using an unpaired, two-tailed Student’s *t* test. For analysis of unequal sample sizes, Welch’s *t* test was employed. Multiple group comparisons were analyzed by one-way analysis of variance (ANOVA), followed by Tukey’s or Bonferroni post hoc correction, as appropriate. Effect size was calculated using Cohen’s d. To validate findings with unbalanced sample sizes, bootstrap confidence intervals (95%) and random subsampling analyses were performed. A *P* value less than 0.05 was considered statistically significant.

## Results

### 5′-tiRNA-Lys is upregulated in the inflamed intestinal epithelium of IBD patients

**Figure 1:**
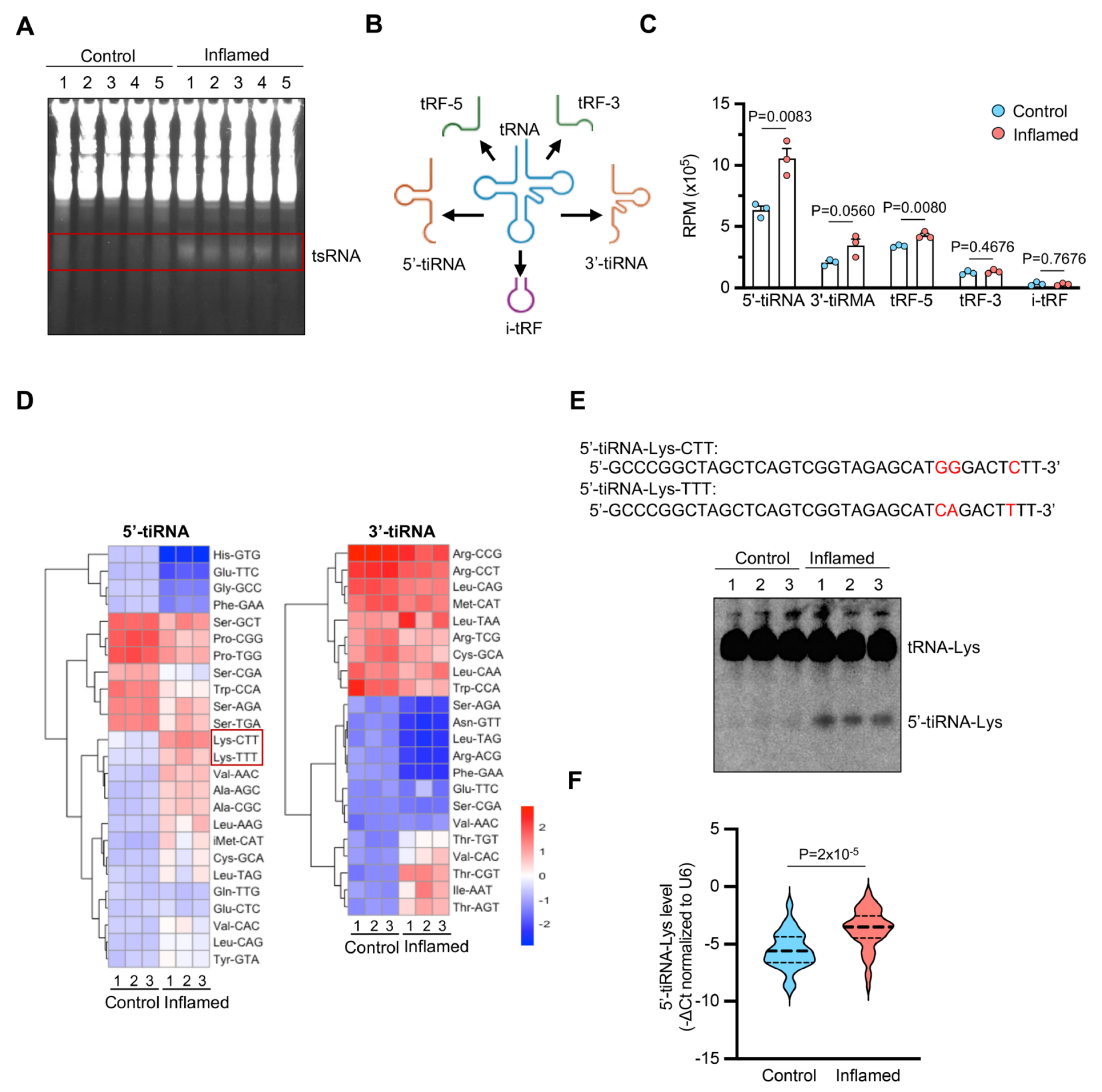
Figure 1 5′-tiRNA-Lys is upregulated in the IECs of IBD patients (A) RNA PAGE analysis of the small RNA profile of IECs isolated from non-inflamed (Control) and inflamed (Inflamed) tissues from IBD patients. The red box highlights the tsRNAs. (B) Schematic classification of tsRNA subtypes on the basis of cleavage positions in mature tRNAs: 5′-tiRNA, 3′-tiRNA, tRF-5, tRF-3, and internal tRF (i-tRF). (C) Comparison of the expression levels of tsRNA subtypes in inflamed vs non-inflamed IECs. n = 3 samples. (D) Heatmap depicting the differential expression of 5′-tiRNAs and 3′-tiRNAs in inflamed vs non-inflamed tissues. 5′-tiRNA-Lys-CTT and 5′-tiRNA-Lys-TTT (red boxes) are markedly upregulated in inflamed tissues. n = 3 samples. (E) Sequence alignment of 5′-tiRNA-Lys-CTT and 5′-tiRNA-Lys-TTT isoforms (top) and Northern blot analysis of 5′-tiRNA-Lys levels. n = 3 samples. (F) qPCR analysis of 5′-tiRNA-Lys levels in intestinal tissues from non-inflamed and inflamed IBD patients. Data are normalized to U6 snRNA. Control: n = 25 samples; inflamed: n = 73 samples. Data are presented as the mean ± SEM. Statistical analysis was performed using two-tailed unpaired t test in (C) and Welch’s t test in (F).

To investigate whether tRNA-derived small RNAs (tsRNAs) are dysregulated in IBD, we isolated the intestinal epithelium from non-inflamed (control) and inflamed mucosal tissues of patients with IBD. RNA PAGE analysis revealed that the tsRNA levels were elevated in the inflamed samples compared with those in the control samples ( Figure 1A). High-throughput sequencing further revealed that among the various tsRNA subclasses ( Figure 1B), 5′-tiRNAs exhibited the most prominent upregulation, followed by 3′-tiRNAs and tRF-5s, whereas tRF-3s and i-tRFs remained unchanged ( Figure 1C).

Focusing on tiRNAs, we observed significantly higher levels of 5′-tiRNA-Lys-CTT and 5′-tiRNA-Lys-TTT in the inflamed samples ( Figure 1D). Given the minimal sequence divergence between these isoforms (differing by three nucleotides; Figure 1E), we selected 5′-tiRNA-Lys-CTT (designated ″5′-tiRNA-Lys″) for further characterization. Northern blot analysis confirmed that this tiRNA was highly upregulated in the intestinal epithelium of inflamed tissues ( Figure 1E). Further qPCR analyses of 25 controls versus 73 inflamed tissues revealed a significant increase in 5′-tiRNA-Lys in the inflamed samples ( *P* = 2 × 10 ^-5^, *t* statistic = –5.05, Cohen’s d = 1.12; Figure 1F). Taken together, these findings suggest that 5′-tiRNA-Lys may contribute to the pathogenesis and progression of IBD by influencing intestinal epithelium function.

### 5′-tiRNA-Lys ameliorates DSS-induced colitis

**Figure 2:**
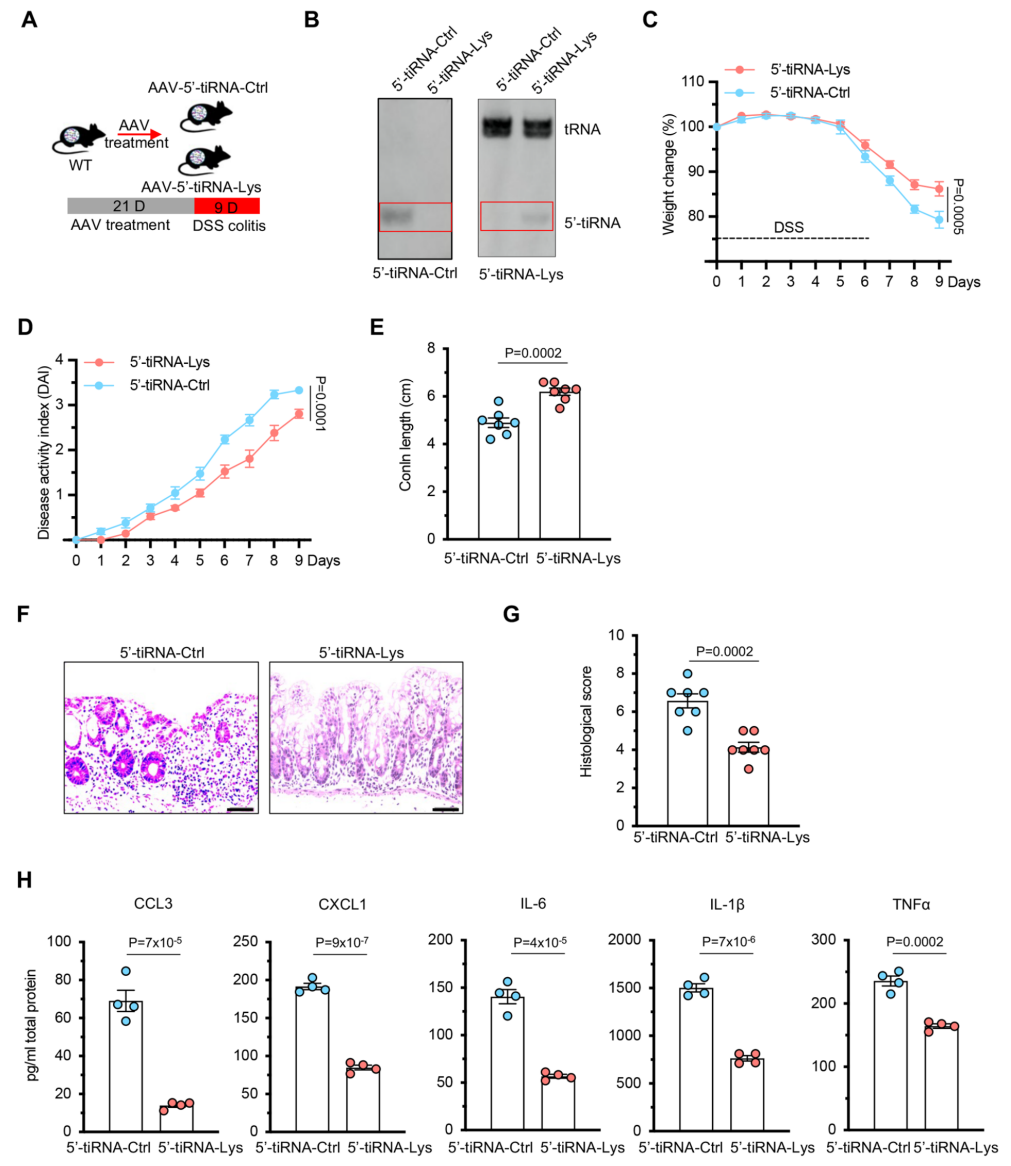
Figure 2 5′-tiRNA-Lys ameliorates DSS-induced colitis (A) Schematic of the experimental design. (B) Northern blot analysis of intestinal tissues post-AAV9 infection confirmed the overexpression of 5′-tiRNA-Lys (vs 5′-tiRNA-Ctrl). (C) Body weight changes during DSS treatment. n = 7 mice. (D) Disease activity index (DAI) scores, including weight loss, stool consistency, and rectal bleeding. n = 7 mice. (E) Colon length measurements post-DSS challenge. (F) Representative H&E-stained colon sections. Scale bar: 50 μm. (G) Histological scores based on epithelial damage, immune infiltration, and crypt loss. (H) Inflammatory cytokine levels (CCL3, CXCL1, IL-6, IL-1β, and TNFα) in colon tissues. Data are presented as the mean ± SEM. Statistical analysis was performed using two-tailed unpaired t tests in (C–H).

To assess the functional significance of 5′-tiRNA-Lys in intestinal inflammation, we used an adeno-associated virus (AAV) vector to overexpress 5′-tiRNA-Lys or a control fragment (5′-tiRNA-Ctrl) in the colons of the mice ( Figure 2A). After 21 days of AAV infection, Northern blot analysis confirmed a significant increase in tiRNA expression in the intestine ( Figure 2B). The mice were then subjected to DSS administration to induce colitis. Compared with control mice, those overexpressing 5′-tiRNA-Lys presented significantly less body weight loss ( Figure 2C) and a lower disease activity index ( Figure 2D). The DSS-induced shortening of the colon was also alleviated in the 5′-tiRNA-Lys overexpression group ( Figure 2E). Histopathological analysis using H&E staining revealed improved epithelial and mucosal integrity in 5′-tiRNA-Lys-treated mice, as evidenced by lower histology scores ( Figure 2F,G). Furthermore, the levels of inflammatory cytokines (CCL3, CXCL1, IL-6, IL-1β, and TNFα) in colon tissue were significantly reduced in mice overexpressing 5′-tiRNA-Lys ( Figure 2H). Collectively, these results suggest that 5′-tiRNA-Lys has protective effects on inflammation in a DSS-induced colitis model.

### 5′-tiRNA-Lys induces autophagy in IECs

**Figure 3:**
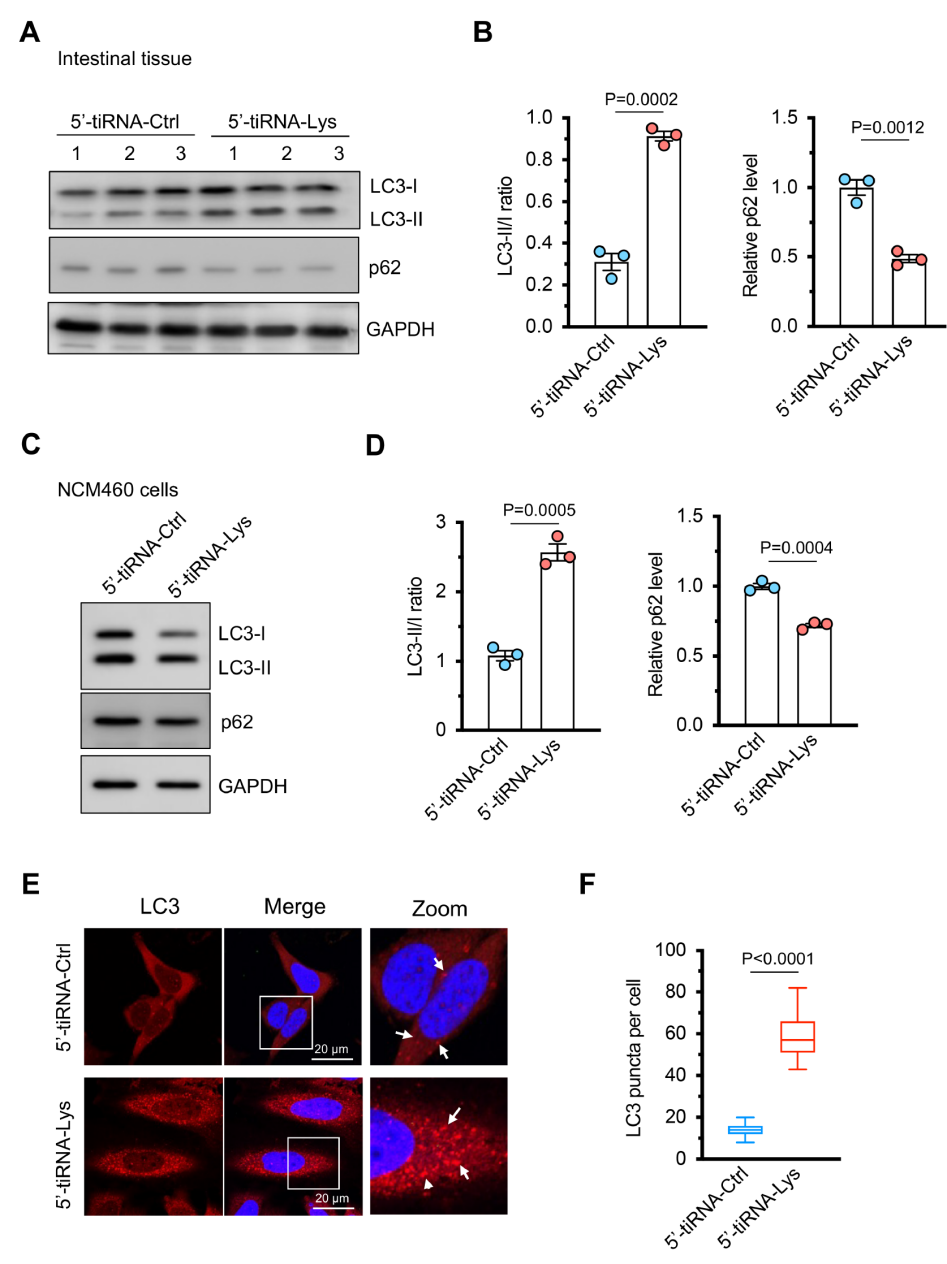
Figure 3 5′-tiRNA-Lys induces autophagy in IECs (A) Western blot analysis of LC3-I/II and p62 protein levels in colonic tissues from mice overexpressing 5′-tiRNA-Lys or the control. GAPDH served as a loading control. (B) Quantification of the LC3-II/LC3-I ratio and p62 protein levels. n = 3 independent experiments. (C) Western blot analysis of LC3-I/II and p62 protein levels in human NCM460 cells transfected with 5′-tiRNA-Lys or the control (5′-tiRNA-Ctrl). GAPDH served as a loading control. (D) Quantification of the LC3-II/LC3-I ratio and p62 protein levels. n = 3 independent experiments. (E) Immunofluorescence staining of LC3 (red) in NCM460 cells transfected with 5′-tiRNA-Lys or the control. The nuclei were counterstained with DAPI (blue). The arrows indicate LC3 puncta. Scale bar: 20 μm. (F) Quantification of the number of LC3 puncta per cell. n = 50 cells per group. Data are presented as the mean ± SEM (***P < 0.001). Statistical analysis was performed using two-tailed unpaired t tests in (B,D,F).

Given the essential role of autophagy in maintaining intestinal barrier integrity and epithelial cell function [4], we next examined whether 5′-tiRNA-Lys regulates IEC autophagy. Western blot analysis of colonic tissues from mice overexpressing 5′-tiRNA-Lys revealed increased levels of the autophagosome-associated protein LC3-II and an elevated LC3-II/LC3-I ratio ( Figure 3A,B). This was accompanied by decreased levels of p62/SQSTM1, an autophagy substrate that is degraded during active autophagic flux ( Figure 3A,B). To further validate these findings, we transfected the normal human intestinal epithelial cell line NCM460 with 5′-tiRNA-Lys and observed similar increases in LC3-II protein levels with concurrent p62 degradation ( Figure 3C,D). Immunofluorescence staining further revealed a pronounced increase in LC3 puncta formation ( Figure 3E,F). These findings suggest that 5′-tiRNA-Lys promotes autophagic flux in IECs, potentially contributing to its protective role in colitis.

### 5′-tiRNA-Lys directly interacts with the RNA-binding protein EWSR1

**Figure 4:**
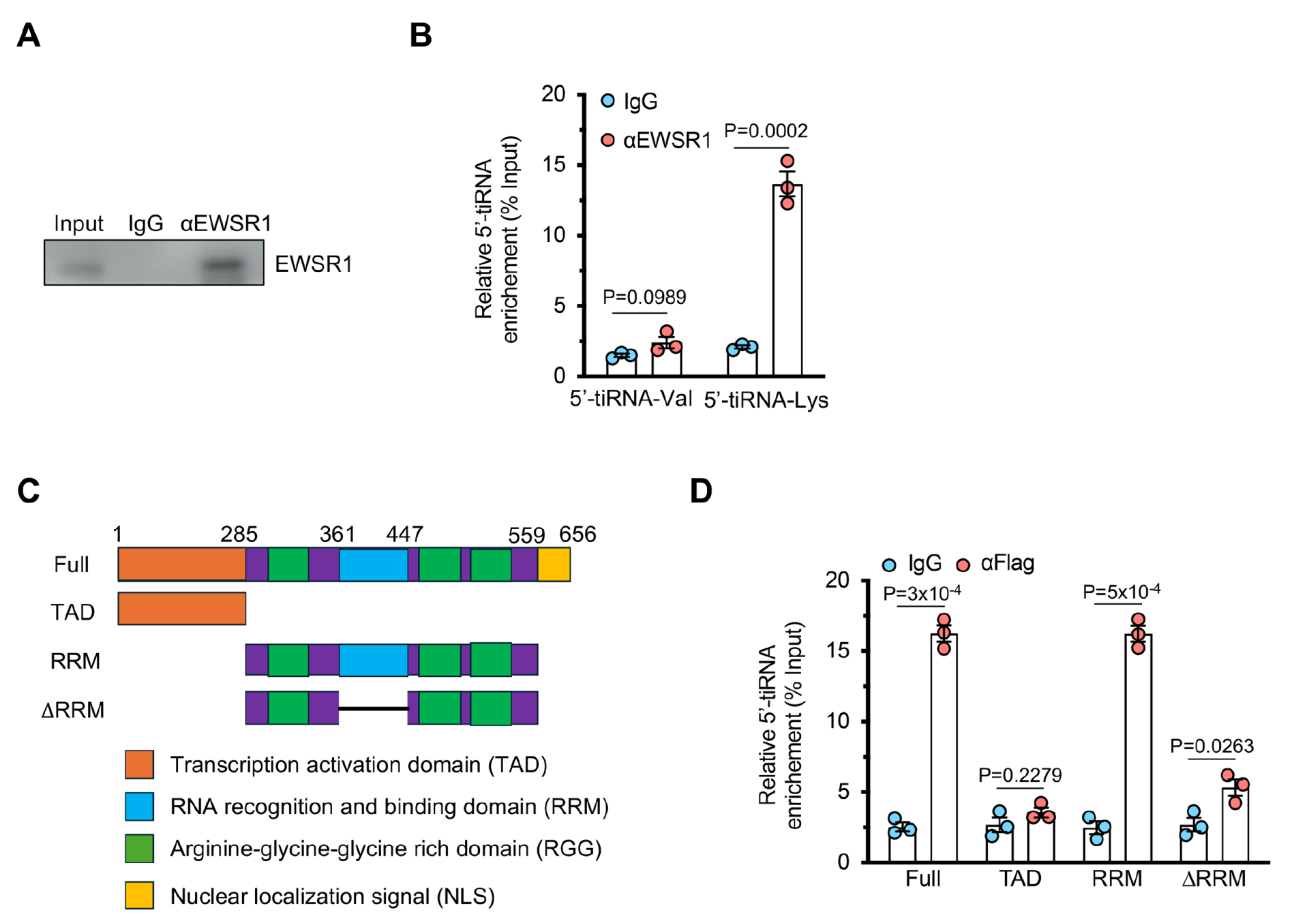
Figure 4 5′-tiRNA-Lys directly interacts with the RNA-binding protein EWSR1 (A) Western blot analysis of the efficiency of EWSR1 protein immunoprecipitation. (B) qPCR analysis of tiRNA enrichment by the EWSR1 antibody. n = 3 independent experiments. (C) Schematic representation of the EWSR1 protein domains. (D) Pull-down analysis of interactions between 5′-tiRNA-Lys and different Flag-tagged EWSR1 domains. n = 3 independent experiments. Data are presented as the mean ± SEM. Statistical analysis was performed using two-tailed unpaired t tests in (B,D).

To elucidate the molecular mechanisms underlying 5′-tiRNA-Lys-mediated autophagy, we first attempted to identify its protein binding partners in NCM460 cells. We thus performed a pull-down assay using biotin-labelled 5′-tiRNA-Lys followed by streptavidin pull-down and mass spectrometry analysis. The results revealed that Ewing Sarcoma breakpoint region 1/EWS RNA binding protein 1 (EWSR1) is a major RNA-binding protein that interacts with 5′-tiRNA-Lys ( Supplementary Table S4). To validate this interaction, we conducted RNA immunoprecipitation (RIP) assays and found that an EWSR1 antibody effectively enriched 5′-tiRNA-Lys but not 5′-tiRNA-Val, a control tiRNA ( Figure 4A,B). Next, we identified the specific domain of EWSR1 responsible for its interaction with 5′-tiRNA-Lys. EWSR1 consists of a transcriptional activation domain (TAD), an RNA recognition and binding domain (RRM), an arginine-glycine-glycine rich domain (RGG), and a nuclear localization signal (NLS) ( Figure 4C) [36]. To pinpoint the region mediating the interaction, we generated Flag-tagged constructs expressing either full-length EWSR1 or various deletion mutants. Pull-down assays using these constructs demonstrated that only the constructs harboring the RNA-binding domain (RRM) could pull down 5′-tiRNA-Lys ( Figure 4D), indicating that the RRM is essential for the interaction between EWSR1 and 5′-tiRNA-Lys.

### 5′-tiRNA-Lys suppresses miR-125a processing

**Figure 5:**
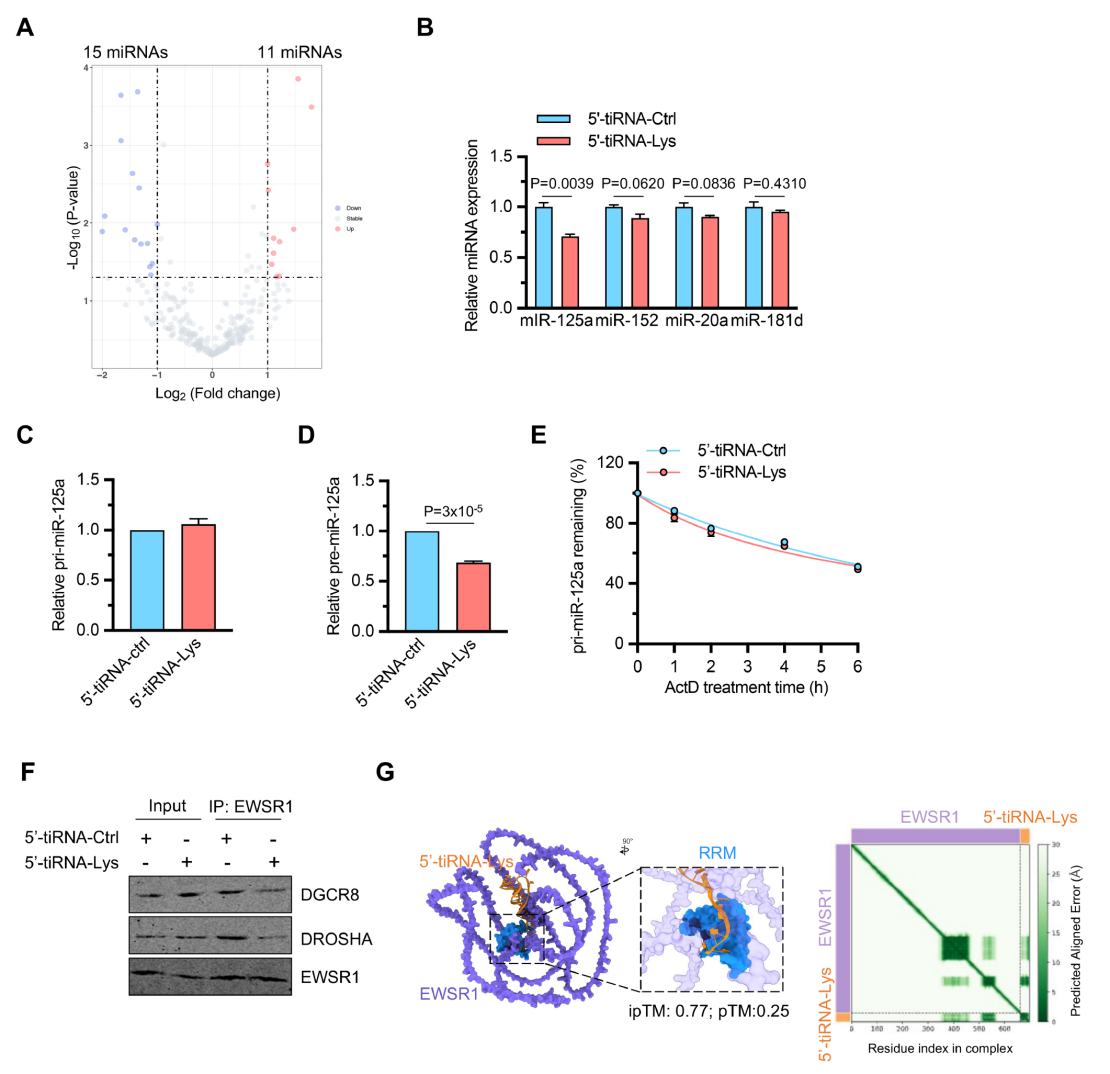
Figure 5 5′-tiRNA-Lys suppresses miR-125a processing (A) Small RNA high-throughput sequencing analysis. (B) qPCR validation of downregulated miRNAs. n = 3 independent experiments. (C,D) qPCR analysis of pre-miR-125a (C) and pri-miR-125a (D). n = 3 independent experiments. (E) RNA stability analysis of pri-miR-125a following actinomycin D (ActD) treatment in cells transfected with 5′-tiRNA-Lys or the control. n = 3 independent experiments. (F) Co-immunoprecipitation assays of DGCR8 with EWSR1 and DROSHA in the presence of 5′-tiRNA-Lys. (G) Computational modelling showing the binding affinity of 5′-tiRNA-Lys and pri-miR-125a for the RRM domain of EWSR1. Data are presented as the mean ± SEM. Statistical analysis was performed using a two-tailed unpaired t test in (B–D).

EWSR1 is known to specifically interact with a large subset of primary microRNAs (pri-miRNAs) by binding to sequences flanking their stem-loop regions, which modulates their processing via the Drosha/DGCR8 microprocessor complex [37]. Given this crucial role of EWSR1 in miRNA biogenesis, we hypothesized that 5′-tiRNA-Lys might influence miRNA maturation. To test this possibility, we first identified 11 upregulated and 15 downregulated miRNAs by miRNA sequencing of NCM460 cells transfected with 5′-tiRNA-Lys ( Figure 5A). Among the downregulated miRNAs, miR-125a, miR-152, miR-20a and miR-181d are known to be involved in autophagy; qPCR analysis confirmed that miR-125a was the most consistently and significantly reduced miRNA ( Figure 5B). Furthermore, we found that 5′-tiRNA-Lys specifically inhibited the levels of the precursor miR-125a (pre-miR-125a) without affecting the primary transcript (pri-miR-125a) ( Figure 5C,D). To exclude the possibility that 5′-tiRNA-Lys affects pri-miR-125a stability, we performed RNA stability assays using actinomycin D treatment. The decay curves for pri-miR-125a were nearly identical between control and 5′-tiRNA-Lys-transfected cells, confirming that the observed reduction in pre-miR-125a was due to impaired processing rather than enhanced degradation of the primary transcript ( Figure 5E). Co-immunoprecipitation assays revealed that overexpression of 5′-tiRNA-Lys disrupted the assembly of the EWSR1-DROSHA-DGCR8 microprocessor complex, thereby reducing the interaction between DGCR8 and DROSHA ( Figure 5F). Protein-RNA complex structure prediction using AlphaFold 3 revealed a direct interaction between 5′-tiRNA-Lys and EWSR1, with 5′-tiRNA-Lys (orange) binding specifically to the RNA recognition motif (RRM, blue) of EWSR1 (purple) ( Figure 5G). The model demonstrated high confidence in the interaction interface, as indicated by the predicted aligned error (PAE) heatmap, with an ipTM score of 0.77. These data suggest that 5’-tiRNA-Lys modulates miR-125a biogenesis by competitively binding to the RRM domain of EWSR1 and disrupting the EWSR1-mediated processing machinery.

### miR-125a targets UVRAG to regulate autophagy under 5’-tiRNA-Lys control

**Figure 6:**
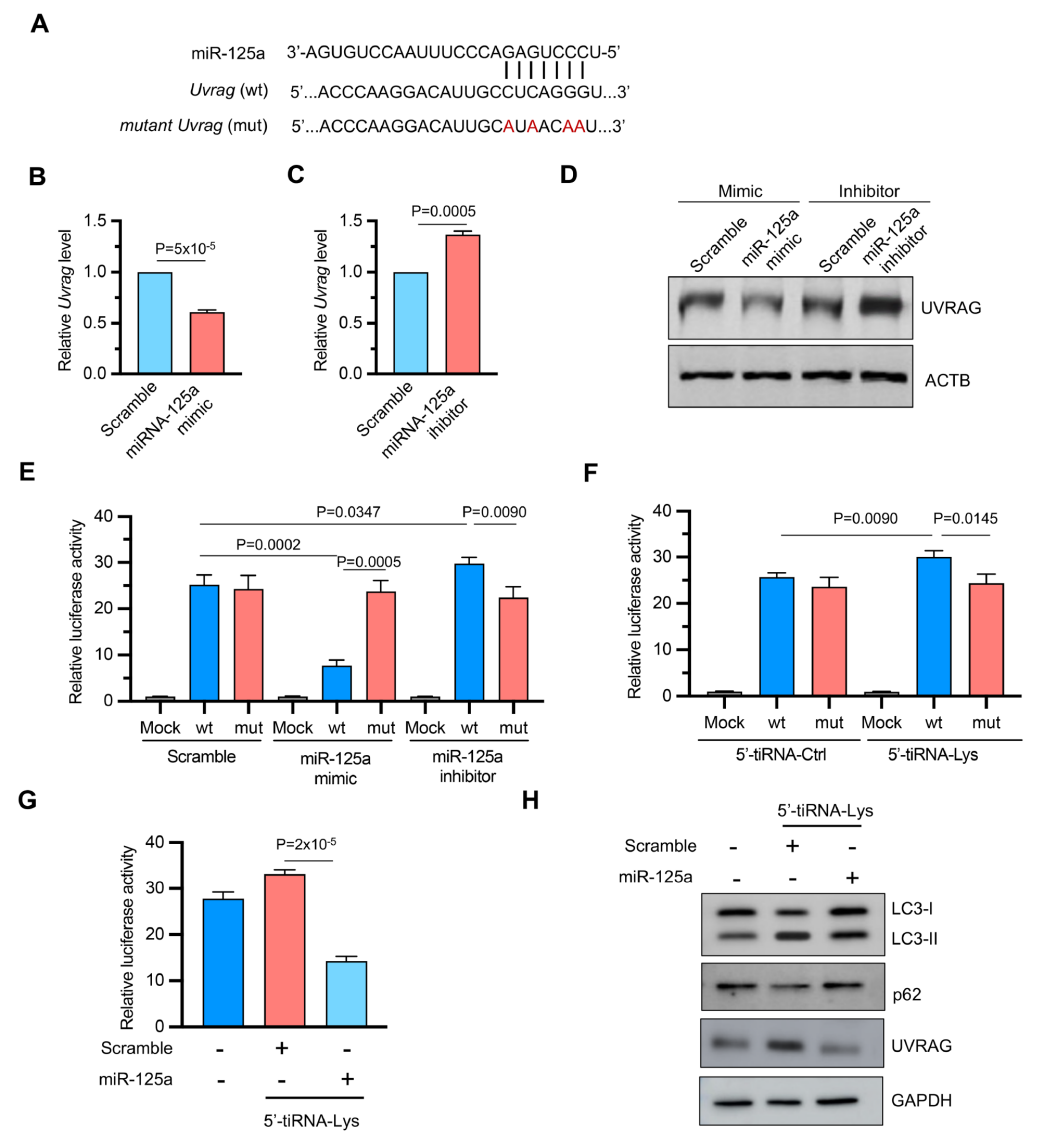
Figure 6 miR-125a targets UVRAG to regulate autophagy under 5′-tiRNA-Lys control (A) Predicted miR-125a binding sites in the 3′UTR of UVRAG mRNA. (B,C) qPCR analysis of UVRAG mRNA levels in NCM460 cells following miR-125a overexpression (B) or inhibition (C). n = 3 independent experiments. (D) Western blot analysis of UVRAG protein levels in NCM460 cells transfected with miR-125a mimics, inhibitor, or respective controls. ATCB served as a loading control. (E) Luciferase reporter assays of wt or mut UVRAG 3′UTR constructs in response to miR-125a manipulation. n = 3 independent experiments. (F) Effect of 5′-tiRNA-Lys on the same luciferase reporter system described in (E). n = 3 independent experiments. (G) UVRAG 3′UTR wt reporter activity in cells co-transfected with 5′-tiRNA-Lys and miR-125a mimics or NC. n = 3 independent experiments. (H) Western blot analysis of the protein levels of autophagy markers (LC3-I/II and p62) and UVRAG in NCM460 cells transfected with scramble control, 5′-tiRNA-Lys alone, or 5′-tiRNA-Lys plus miR-125a. GAPDH served as a loading control. Data are presented as the mean ± SEM. Statistical analysis was performed using a two-tailed unpaired t tests in (B,C) and one-way ANOVA in (E–G).

To understand how a reduction in miR-125a leads to increased autophagy, we explored potential target genes of miR-125a that are involved in autophagy and identified *UVRAG* as a likely candidate [38]. Bioinformatics analysis using TargetScan predicted a binding site for miR-125a in the 3’UTR of *UVRAG* ( Figure 6A). The manipulation of miR-125a levels in NCM460 cells confirmed this relationship: overexpression of miR-125a suppressed both the mRNA and protein levels of UVRAG, whereas the inhibition of miR-125a increased UVRAG expression ( Figure 6B–D). In luciferase reporter assays, miR-125a bound to the wild-type (wt) but not the mutated (mut) *UVRAG* 3′UTR, leading to reduced luciferase activity ( Figure 6E). Importantly, 5′-tiRNA-Lys overexpression increased UVRAG reporter activity, and this effect was reversed by restoring miR-125a levels ( Figure 6F,G). To directly assess the functional consequences of this regulatory axis on autophagy, we performed western blot analysis of key autophagy markers. 5′-tiRNA-Lys treatment significantly increased UVRAG protein expression, increased LC3-II level and reduced p62 accumulation, whereas co-transfection with miR-125a reversed these effects, restoring UVRAG to baseline level while normalizing the levels of autophagy markers ( Figure 6H). These findings suggest that by downregulating miR-125a processing, 5′-tiRNA-Lys promotes UVRAG expression and enhances autophagy in IECs.

## Discussion

In this study, we elucidate a previously unrecognized role for 5′-tiRNA-Lys in maintaining intestinal epithelial homeostasis through its interaction with the RNA-binding protein EWSR1 and subsequent regulation of miRNA biogenesis. This process plays a critical role in the pathogenesis and progression of IBD, as evidenced by the protective effects of 5′-tiRNA-Lys overexpression in mitigating colitis and restoring autophagy. Our findings not only advance the mechanistic understanding of tsRNAs in orchestrating cellular stress responses but also highlight 5’-tiRNA-Lys as a novel therapeutic candidate for IBD.

The discovery that 5′-tiRNA-Lys suppresses miR-125a maturation by disrupting the interaction of EWSR1 with the Drosha/DGCR8 complex underscores a noncanonical regulatory axis linking tsRNAs to miRNA processing. This mechanism expands the functional repertoire of tsRNAs beyond their established roles in translation control or mRNA stability [ 16, 24, 28] , positioning them as dynamic modulators of RNA‒protein interactions. The specificity of 5′-tiRNA-Lys for miR-125a—a miRNA directly targeting UVRAG, a key autophagy promoter—reveals how localized RNA interference can amplify protective cellular responses during inflammation. These observations align with emerging evidence that UVRAG deficiency exacerbates intestinal barrier dysfunction [ 39, 40] ; however, our work uniquely connects tRNA-derived molecules to this pathway.

The interaction between 5′-tiRNA-Lys and EWSR1’s RRM domain provides mechanistic insight into how tsRNAs influence nuclear RNA processing. EWSR1’s role in recruiting Drosha/DGCR8 to specific pri-miRNAs [37] suggests that 5′-tiRNA-Lys acts as a molecular decoy, sequestering EWSR1 and preventing its engagement with miR-125a precursors. Such interference with microprocessor function may explain the selective suppression of miR-125a, as structural features of its pri-miRNA stem-loop likely render its processing uniquely dependent on EWSR1. However, a comprehensive analysis of all EWSR1-dependent miRNAs affected by 5′-tiRNA-Lys warrants further investigation, as this could reveal additional regulatory pathways relevant to intestinal homeostasis and inflammatory responses beyond the autophagy mechanism we have characterized.

While our data strongly support UVRAG-mediated autophagy as a primary mechanism by which 5′-tiRNA-Lys ameliorates colitis, additional pathways may contribute to its anti-inflammatory effects. Our findings suggest potential cross-talk between autophagy and inflammatory signaling cascades, including the NF-κB and MAPK pathways. Enhanced autophagy is known to suppress NF-κB activation through selective degradation of signaling intermediates [41], and the significant reduction in inflammatory cytokines observed in our mouse model ( Figure 2H) is consistent with both autophagy-dependent and potentially autophagy-independent mechanisms. Future studies will be valuable in dissecting these complementary pathways and their relative contributions to the protective effects of 5′-tiRNA-Lys in IBD.

The upregulation of 5’-tiRNA-Lys in the intestinal epithelium of IBD patients and its protective effects in reducing colitis highlight its potential as both a diagnostic biomarker and therapeutic target. Our previous work demonstrated that ANG, the enzyme responsible for tiRNA generation, is upregulated during early IBD stages [ 29, 30] , suggesting that 5′-tiRNA-Lys induction may serve as an early protective response to inflammation. Therefore, quantifying the levels of 5′-tiRNA-Lys and ANG in intestinal biopsies may be valuable biomarkers for IBD diagnosis. However, the dynamic expression of 5′-tiRNA-Lys during chronic inflammation remains unclear. Future studies that track its levels across IBD phases—from active inflammation to remission—are necessary to evaluate its potential for predicting disease progression and relapse. Therapeutically, restoring the activity of 5′-tiRNA-Lys in IECs represents a novel approach to augment autophagy and suppress inflammation. While our DSS-induced colitis model shows promising results, translating these findings to human IBD patients requires additional investigation, particularly regarding long-term efficacy and delivery strategies for chronic disease management. Our findings, along with the growing success of RNA-based therapies for gastrointestinal disorders [ 42, 43] , support the possibility that synthetic 5′-tiRNA-Lys mimics or ANG activators could serve as promising therapeutic interventions.

In summary, our study reveals a tsRNA-driven regulatory circuit that fine-tunes intestinal epithelial integrity through EWSR1-dependent miRNA processing. By bridging tRNA fragmentation products with autophagy modulation, these findings provide a framework for developing RNA-based interventions to restore epithelial homeostasis in IBD.

## Supporting information

25113

## Supplementary Data

Supplementary data is available at *Acta Biochimica et Biophysica Sinica* online.
