# Supplementary material for: 5′-tiRNA-Lys maintains intestinal epithelial homeostasis by EWSR1-dependent suppression of miR-125a and autophagy activation: 5′-tiRNA-Lys modulates autophagy in IECs
Source: Acta Biochim Biophys Sin (Shanghai). 2025 Jun 23;58(2):290–302. doi: 10.3724/abbs.2025074 (PMC12900774; doi:10.3724/abbs.2025074)
Supplement: 25113 [file 25113.Supplementary_tables.docx]

**Supplementary Table S1. Probes for Northern blot analysis**

| **Northern blot probe** | **Sequence (5**′ **to 3**′**)** |
| --- | --- |
| 5′-tiRNA-Ctrl-DIG | (DIG)-GCATGAGCAGATTCTGCCCTCACGTGACTCACA |
| 5′-tiRNA-Lys-DIG | (DIG)-GAGTCCCATGCTCTACCGACTGAGCTAGCCGG |

**Supplementary Table S2. Sequences of primers for qPCR**

| **Name** | **Sequence (5**′ **to 3**′**)** |
| --- | --- |
| 5′-tiRNA-Lys RT | GTCGTATCCAGTGCAGGGTCCGAGGTATTCGCACTGGATACGACAAGAGT |
| 5′-tiRNA-Lys F | GCCGCCCGGCTAGCTCAGT |
| 5′-tiRNA-Lys R | GTGCAGGGTCCGAGGT |
| 5′-tiRNA-Lys Probe | CTGGATACGACAAGAGTCC |
| 5′-tiRNA-Val RT | GTCGTATCCAGTGCAGGGTCCGAGGTATTCGCACTGGATACGACAGGCGA |
| 5′-tiRNA-Val F | GCCGTTTCCGTAGTGTAGT |
| 5′-tiRNA-Val R | GTGCAGGGTCCGAGGT |
| 5′-tiRNA-Val- Probe | CTGGATACGACAGGCGAAC |
| U6 primer F | CTCGCTTCGGCAGCACA |
| U6 primer R | AACGCTTCACGAATTTGCGT |
| U6 primer Probe | CCATGCTAATCTTCTCTGTATCGTTCC |
| miRNA-125a RT | GTCGTATCCAGTGCAGGGTCCGAGGTATTCGCACTGGATACGACTCACAG |
| miRNA-125a F | GCCCTCCCTGAGACCCTTAA |
| miRNA-125a R | GTGCAGGGTCCGAGGT |
| miRNA-125a Probe | CTGGATACGACTCACAGGT |
| miRNA-152 RT | GTCGTATCCAGTGCAGGGTCCGAGGTATTCGCACTGGATACGACCCAAGT |
| miRNA-152 F | GCCTCAGTGCATGACAGAAC |
| miRNA-152 R | GTGCAGGGTCCGAGGT |
| miRNA-152 Probe | CTGGATACGACCCAAGTCT |
| miRNA-20a RT | GTCGTATCCAGTGCAGGGTCCGAGGTATTCGCACTGGATACGACCTACCT |
| miRNA-20a F | GCCTAAAGTGCTTATAGT |
| miRNA-20a R | GTGCAGGGTCCGAGGT |
| miRNA-20a Probe | CTGGATACGACCTACCTGC |
| miRNA-181d RT | GTCGTATCCAGTGCAGGGTCCGAGGTATTCGCACTGGATACGACACCCAC |
| miRNA-181d F | GCCAACATTCATTGTTGT |
| miRNA-181d R | GTGCAGGGTCCGAGGT |
| miRNA-181d Probe | CTGGATACGACACCCACCG |
| *UVRAG* F | TGACAATTCGTTGCAGGCAGTTA |
| *UVRAG* R | AGGCAACTTGACACCGCATACA |
| *ACTB* F | CACCATTGGCAATGAGCGGTTC |
| *ACTB* R | AGGTCTTTGCGGATGTCCACGT |

**Supplementary Table S3. Oligonucleotide sequences**

| **Name** | **Sequence (5**′ **to 3**′**)** |
| --- | --- |
| 5′-tiRNA-Ctrl | (PO4)-UGUGAGUCACGUGAGGGCAGAAUCUGCUCAUGC |
| 5′-tiRNA-Lys-CTT | (PO4)-GCCCGGCUAGCUCAGUCGGUAGAGCAUGGGACUCUU |
| 5′-tiRNA-Val-AAC | (PO4)-GUUUCCGUAGUGUAGUGGUUAUCACGUUCGCCU |
| miR-125a mimic | UCCCUGAGACCCUUUAACCUGUGA |
| miR-125a inhibitor | UCACAGGUUAAAGGGUCUCAGGGA (2'-OMe-RNA) |
| Scramble control | UUGUACUACACAAAAGUACUG (2'-OMe-RNA) |

**Supplementary Table S4. The top 10 interacting proteins of 5**′**-tiRNA-Lys**

| **No** | **ID** | **Gene name** | **Gene symbol** | **Score** | **Coverage** | **#Aas** | **MW (kDa)** |
| --- | --- | --- | --- | --- | --- | --- | --- |
| 1 | H7BY36 | Ewing sarcoma breakpoint region 1 | EWSR1 | 85.55 | 14.87 | 656 | 68.5 |
| 2 | H3BNC1 | RNA binding motif protein, X-linked | RBMX | 77.84 | 35.14 | 37 | 4 |
| 3 | K7ENG2 | U2 small nuclear RNA auxiliary factor 2 | U2AF2 | 67.37 | 5.86 | 307 | 33.9 |
| 4 | C9J5V9 | Y-box binding protein 1 | YBX1 | 63.65 | 3.7 | 216 | 23.6 |
| 5 | Q01081 | U2 small nuclear RNA auxiliary factor 1 like 5 | U2AF1L5 | 50.67 | 19.16 | 167 | 19.7 |
| 6 | P51991 | Heterogeneous nuclear ribonucleoprotein A3 | HNRNPA3 | 47.12 | 3.65 | 356 | 37 |
| 7 | F8WJN3 | Cleavage and polyadenylation specific factor 6 | CPSF6 | 40.1 | 5.23 | 478 | 52.2 |
| 8 | D6RF44 | Heterogeneous nuclear ribonucleoprotein D | HNRNPD | 26.99 | 21.62 | 111 | 12.5 |
| 9 | O43809 | Nudix hydrolase 21 | NUDT21 | 35.14 | 16.3 | 227 | 26.2 |
| 10 | G3V3U4 | Proteasome subunit alpha 6 | PSMA6 | 31.25 | 11.21 | 107 | 11.6 |
